# Development of a Hospital-Stakeholder Collaboration Tool Using Mixed Methods to Assess Stakeholder Perspectives for Hospital Service Improvement

**DOI:** 10.4314/ejhs.v33i6.18

**Published:** 2023-11

**Authors:** Purwaningsih Purwaningsih, Nasronudin Nasronudin, Nyoman Anita Damayanti, Mahmudah Mahmudah, Sri Andarini, Bagus Qomarudin, Djazuly Chalidyanto, Slamet Riyadi Yuwono, Aby Nugrah Septanto, Hakim Zulkarnain

**Affiliations:** 1 Faculty of Public Health, Universitas Airlangga, Surabaya, Indonesia; 2 Faculty of Nursing, Universitas Airlangga, Surabaya, Indonesia; 3 Faculty of Medicine, Universitas Airlangga, Surabaya, Indonesia; 4 Faculty of Public Health, Universitas Airlangga, Surabaya, Indonesia; 5 Faculty of Medicine, Universitas Brawijaya, Malang, Indonesia; 6 Department of Nutrition, Polytechnic of Health Ministry of Health Surabaya, Surabaya, Indonesia; 7 Faculty of Sport Science, State University of Surabaya, Indonesia

**Keywords:** Hospital-stakeholder, Patient Feedback, Service Improvement, Patient Experience, Coverage

## Abstract

**Background:**

The purpose of this study was to develop the Hospital-Stakeholder Collaboration (HSC) Tool and Hospital Performance Factor (HPF) Tool to explore stakeholder perception and value for hospital service improvement.

**Methods:**

This exploratory mixed-method study involved three steps: initial tool development (Step 1), validity testing (Step 2), and module development (Step 3). In Step 1, qualitative data collection through literature reviews, focus group discussions, and interviews with hospital management experts led to the creation of the preliminary tools. Step 2 involved qualitative analysis by α 5-member expert panel, followed by quantitative analysis with 36 respondents for validity (Pearson correlation, α = 0.05) and reliability (Cronbach's Alpha, α = 0.6) tests. Step 3 encompassed the final module development.

**Results:**

The HSC tool contains 6 domains and the HPF tool contains 4 perspectives. The 6 HSC domains were: 1) stakeholder identification, 2) interactive dialogue, 3) commitment, 4) planning, 5) implementation, 6) change in action and behavior. The 4 HPF perspectives were: 1) stakeholder perspective, 2) financial perspective, 3) internal business process, and 4) staff and organizational capacity. The values of the HSC tool validity and reliability tests were around 0,0046 and around 0,995, respectively. Additionally, the values of the HPF tool validity and reliability tests were around 0,0062 and around 0,995, respectively.

**Conclusion:**

This study offers a practical tool for needs assessment for the improvement of service by analyzing direct feedback from hospital stakeholders and measuring hospital performance factors.

## Introduction

This study focused on service improvement, which is another side of quality improvement. The quality improvement focused on the clinical processes, and the service improvement focused on the non-clinical aspects, which led to a more satisfying and patient-centered experience (1–3). The future of the hospital proposed by Filho contains a pillar of patient-centered care. It includes hospital development that encourages hospital stakeholder collaboration (4,5). To name a few of the hospital-stakeholder groups: patient, patient's family, staff (i.e., doctors, nurses, midwives, security staff, etc.), and insurance company (6).

The majority of hospital stakeholders in Indonesia are only partially involved. For example, the patient's opinion of service satisfaction is simplified into a number and left out of the overall opinion. The stakeholder was only allowed to answer closed questions, and no detailed description or opinion was collected. Therefore, hospital service improvements are mainly based on complaints. This leads to a waste of potential from stakeholder collaboration for the development of hospital performance (6–8).

Hospital service improvement according to patient satisfaction currently measured from the patient's perspective and value in seeking medical help (8). America's survey of the state of health care involved 5,017 patients, 687 doctors, and 521 hospital managers. Service quality is the most important value for doctors and patients, making up 68%-88% of the total value component. Sequentially, the important values for patients are cost (26%) and service (12%). Unfortunately, patient experience only makes up 7% of the value component from the point of view of doctors, and cost (5%) is the last component. Hospital managers say that the most dominant value is employee satisfaction (43%), followed by cost (37%), and employee productivity (20%) (9). The variability of the results of this survey shows differences in values between stakeholders, which makes improving service quality even more challenging without stakeholder collaboration.

The quality of hospital services can be improved by focusing on efficiency, effectiveness, and user experience (9). Innovation based on the synchronization between patients, families, doctors, insurance, and hospital management is complex (8). One example of existing innovation in hospitals for service improvement is cluster analysis. Cluster analysis is the division of service rooms according to the patient's specific needs, for example, the Emergency Department (ED), geriatric care, children's care, oncology care, etc. (10,11). The ED promotes patient streaming, which involves a set of care processes designed for emergency room patients, especially for low-priority patients (9,12). This innovation is based on the input from patients that low-priority patients tend to receive treatment at the end of the long queue (13). The fast-track speeded up low-priority patients treatment, thereby reducing waiting time in the emergency room (13,14) and resulted in satisfied patients (15).

Unfortunately, the development of stakeholder collaboration-based hospitals in Indonesia is still limited, let alone focused only on patient needs (7,8,10,16). There is no system that meets the hospital management and the patient to discuss service improvement, which makes it hard to define value, quality, service, and cost of patient (16). The Indonesian Hospital mainly focused on quality improvement measures such as patient severity, urgency of action, and likelihood of adherence, while service improvement measures such as patient preferences and values have not been considered (17). The limited stakeholder groups that are involved in Quality Improvement (QI) processes, such as those focused only on physician inputs, reportedly do not provide much improvement (18), recruiting a percentage of hospital staff and senior managers into formally organized QI teams is associated with better values on quality indicators and better decision-making (19,20). Since there is no system developed to assess the patient's needs for detailed service improvement, then there is no tool designed for this. The study found that there was no tool had been developed to assess stakeholder needs for service improvement in the OPD setting. This study resolves the gap by developing a hospital-stakeholder collaboration tool and a hospital performance factor tool to explore stakeholder perception and value for hospital service improvement based on need assessment. This instrument will be tested for its validity, and reliability for wider stakeholder-hospital populations.

## Material and Methods

**Figure 1:**
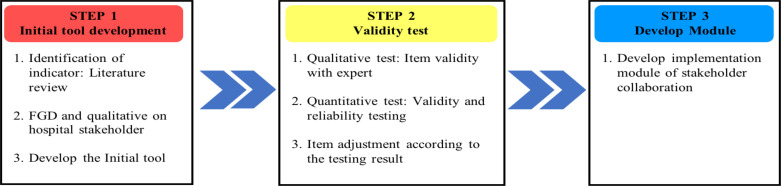
The methodological development framework of the hospital-stakeholder collaboration tool

This study was designed as an exploratory mixed-method study to develop five measurement tools of hospital stakeholder-collaboration. The exploratory step started with exploring the domains of HSC and HPF qualitatively and conducted literature review. Then those domains were used to form the hypothesis and questionnaire, which were tested quantitatively (21). The five tools are HSC (1) for the patient (2) for the patient's family, (3) for internal doctors, (4) for external doctors, and (5) health insurance company. Methodological development was conducted in three steps (Fig 1): (1) initial tool development; (2) validity testing and item adjustment; and (3) module development. Several research methods were employed, including literature reviews, primary qualitative and quantitative research, item consolidation with experts, and peer review by key stakeholders, as described in the following sections (22). The purpose of this study was to develop the Hospital-Stakeholder Collaboration (HSC) Tool and Hospital Performance Factor (HPF) Tool to explore stakeholder perception and value for hospital service improvement. This study was granted ethical clearance by the ethical commission of the Faculty of Nursing, Universitas Airlangga, with the registered number 2749-KEPK dated February 13, 2023. The research steps are illustrated in Figure 1.

**Step 1: Initial tool development**: This step aimed to explore the variables that build hospital-stakeholder collaboration. Identification of indicators. Literature review, qualitative interviews with hospital stakeholders, and development of the initial tool constituted this step.

### 1. Domain and indicator: literature review

**Figure 2:**
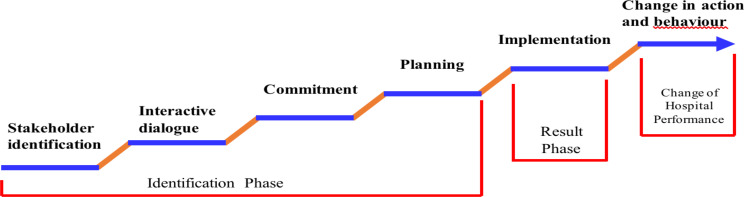
The 6 domains of Hospital Stakeholder Collaboration

The evidence-based typology from a literature review was used as the foundation of the structure and domains of the tool. This review was based on the PICO framework (Table 1). The databases employed were Google Scholar, Scopus, and PubMed. Additional manual research was employed. The search resulted in 150 articles collected; after read the title and abstract, 42 articles were found; and finally, 22 articles contained the related variables and items. The review resulted in the 6 domains of hospital-stakeholder collaboration, which consisted of stakeholder identification, interactive dialogue, commitment, planning, and implementation. Regarding the hospital performance factor, it consisted of stakeholder perspective, financial perspective, internal business process perspective, staff perspective, and organizational capacity perspective (Fig 2).

**Table 1:** PICO framework

Item	Keywords
**P - Patient**	Hospital stakeholder, patient, doctor
**I - Intervention**	Collaboration, Assessment
**C - Comparison**	Tool, Questionnaire
**O - Outcome**	Quality Improvement

### 2. Interviews with hospital stakeholders to confirm the domain and indicator found

Focus Group Discussion (FGD) was conducted by inviting stakeholder representatives for socialization and discussing the initial model of hospital-stakeholder collaboration based on the results of the literature review. The stakeholders involved were 30 people from patients, patient families, internal doctors, external doctors, and insurance company staff, which were distributed equally. The FGD started by presenting the domain from the literature review and asking the panel about the relevance of the domain in terms of hospital service.

The FGD discussed each domain and confirmed the accuracy of its application in hospital business processes. All stakeholders provided input and agreed with the domain on the proposed stakeholder-hospital model. Next, a qualitative study was carried out with stakeholders about the items that make up each indicator. The results of the qualitative interviews were transcribed, analyzed thematically, and inventoried for each indicator to form an initial questionnaire.

### 3. Developing the initial tool

The initial tool was developed by carrying out a suitability analysis for each item that had been inventoried for each indicator. This stage was carried out by researchers and two other experts in the field of hospital management. The distribution of items in each tool is shown in Table 2. The results of this stage were continued in the second stage, namely validity testing.

**Table 2:** Validity and reliability test

Tool	Target population	Item pre-adjustment	Adjustment Action	Validity test (Average ± Std Dev Min - Max)	Interpretation* α = 0.05	Reliability test	Interpretation** α = 0.6
Hospital	Patient	50	Excluded 2 items	0,004 ±	47	0,992	Reliable
Stakeholder			Merged 2 items	0,007	items valid		
Collaboration			into 1	0 – 0,039			
	Patient Family	50	Excluded 2 items	0,001 ±	47	0,997	Reliable
			Merged 2 items	0,004	items valid		
			into 1	0 – 0,021			
	Internal Doctor	40	Excluded 2 items	0,004 ±	38	0,994	Reliable
				0,007	items valid		
				0 – 0.030			
	External Doctor	40	Excluded 2 items	0,003 ±	38	0,995	Reliable
				0,009	items valid		
				0 – 0,038			
	Insurance Company	40	Excluded 2 items	0,011 ±	38	0,997	Reliable
				0,011	items valid		
				0 – 0,044			
		**AVERAGE**		**0,0046**		**0,995**	
Hospital	Patient	45	Excluded 4 items	0,003 ±	41	0,992	Reliable
Performance				0,007	items valid		
Factors				0 - 0,042			
	Patient Family	45	Excluded 4 items	0,003 ±	41 items valid	0,997	Reliable
				0,008			
				0 - 0,048			
	Internal Doctor	35	Excluded 1 items	0,006 ±	33	0,994	Reliable
			Merged 2 items	0,012	items valid		
			into 1	0 - 0,048			
	External	30	Excluded 1 item	0,003 ±	29	0,995	Reliable
	Doctor			0,006	items valid		
				0 - 0,026			
	Insurance	40	Merged 2 items	0,016 ±	39	0,997	Reliable
	Company		into 1	0,015	items valid		
				0 - 0,045			
	**AVERAGE**			**0,0062**		**0,995**		

Note: *Validity test p ≤ α (α = 0.05); *Reliability test p > α (α = 0.6)

### Step 2: Validity testing

This step aimed to check whether the tool passed qualitative and quantitative tests. This step started with a qualitative test by consolidating the item with five expert panels of hospital management. The qualitative interview with the expert was conducted by presenting the initial tool. The expert panel reviews the question from the beginning to the end and supplies feedback on the context and grammar of each question. The test continued with quantitative test by employing the validity and reliability test on the target respondent. Finally, this step ended with an item adjustment according to the testing result.

#### 1. Qualitative test: item validity with experts

The qualitative item validity was determined by recruiting five experts in hospital management. There were several eligibility criteria for the expert: 1) at least 10 years of experience in the field; and 2) being currently part of hospital management. The tools were sent to each expert to request input and corrections regarding the indicator and item of each domain. The expert also requested comments on the item's narration. Finally, to avoid bias, the expert also gave narrative comments on each tool.

#### 2. Quantitative test: validity and reliability testing

The quantitative validity and reliability testing was performed by the recruited target respondent of each tool. The respondents recruited should meet these criteria: the patient should be an active patient of the outpatient department and suffer from chronic disease; the family should actively accompany the patient for the health visit; both of them should have ever visited another outpatient department of a different hospital.

The internal doctor should be legally registered as hospital staff; the external doctor should be a doctor who is working at that hospital but is not a registered staff member. The difference between the two was that the external doctor was a visiting doctor, so they could compare the hospital service with that of a different hospital. The insurance company should have at least three active years with the hospital.

In total, 36 respondents were recruited, including 13 patients, 11 patient families, 5 internal doctors, 4 external doctors, and 3 insurance companies. The target respondents were requested to fill out the tool, and the results were statistically analyzed using SPSS 26 software. The validity of each item was tested by Pearson correlation with α = 0.05. The reliability test for each tool was tested by Cronbach's alpha with α = 0.6.

#### 3. Item adjustment according to the testing result

This step ended with item adjustments according to the qualitative and quantitative tests. The adjustments include: revising the narration of each item, reordering the item, and selecting the item. The item that had a low relevance and low validity score was eliminated. Some items were merged that have similar meanings. The tool is articulated to be understood by the patient and patient family. The final decision is based on the structure and items achieved by consensus among the researchers. This step finalized the design of the tool and made it ready for pilot testing.

### Step 3: Developing the module

The final instrument became the base for module development. The purpose of the module was to guide the manager and the staff of the hospital to collaborate with stakeholders. It contained the step-by-step undertaking of hospital stakeholder-collaboration. The module contained the six domains of hospital-stakeholder collaboration and four perspectives on hospital performance factor. The module was named “Implementation of the Hospital's-Stakeholder Collaboration Module”.

## Results

Data were collected from March 13^th^ to 24^th^, 2023, at Universitas Muhammadiyah Malang Hospital, Indonesia. This section outlines the domain of each tool, the validity test, and the operation of the module. All of this information is available in the module, which could then be used by the nurse or hospital administrative staff to do the needs assessment of the hospital stakeholder. All of the service improvement processes last for 8 weeks.

### Domain

***1. Hospital stakeholder collaboration tool:*** The tool contains six domains that consist of stakeholder identification, interactive dialogue, commitment, planning, implementation, change in action, and behavior. Stage 1, in week 1, involves all stakeholders by using questionnaires to conduct structured interviews so that potential stakeholders are identified to be involved in need assessment (10, 21). In week 1, an interactive dialogue was conducted using questionnaires to obtain information about the ease of obtaining information (22,23), the willingness of stakeholders to conduct joint discussions, the clarity of the information obtained, the language used by officers (24), and the attitude of officers towards patients (25).

The second week was filled with FGDs to discuss the results of the need assessment by bringing together stakeholders and hospital staff representatives. FGDs were measured using questionnaires to structure questions. Starting with the FGD to build the commitment (24,27) of stakeholders; namely, by understanding their respective roles (26,28) and approving service improvement efforts (13,28–30). It is followed by FGD, to build common goals (31,32), to find the goals of service improvement (31,32) and to understand the goals that have been set (33,34). Next, in the second week, the FGD dissemination of results was carried out; namely, reporting the list of perspectives and need assessments of stakeholders (31,32). The list was discussed with experts; namely, appointed hospital senior officers. The end of the second week saw the establishment of a comprehensive Point of Action (POA) agreement on service improvement (39).

The third week focused on the initiation of service improvement through action and behavioral change in business services by OPD (34,35). Service improvement implementers are staff at OPD who refer to the improvement implementation guide containing POA. Focus on service improvement from the third to the eighth weeks so that it can be evaluated in the eighth week.

***2. Hospital performance factor tool.*** In week eight, identified stakeholders evaluated the service improvement process through questionnaires, covering stakeholder, financial, internal business process, and staff and organizational capacity perspectives. Interviews gather their subjective insights, including service wait times and satisfaction. This involves aspects like registration, queue times, and non-medical facilities. Stakeholder satisfaction spans information access, registration, staff friendliness, and feedback avenues. (39.^40^).

The financial perspective domain seeks patients' and families' financial views on payment process, costing, ease of payment, speed, cost appropriateness, and hidden fees presence (33,36). The internal business process domain aims to understand stakeholder perspectives on service speed, staff competence, service quality, and infrastructure quality. Stakeholders evaluated the impact of automated systems and website accessibility on their experience. Additionally, meticulous staff responsiveness is highly regarded. Stakeholders also assess the availability and quality of facilities and infrastructure, encompassing factors like cleanliness, advanced equipment, and therapeutic services (39.^40^).

The last domain is about staff and organizational capacity, which includes competitive HR, a healthy organization and the implementation of the best service governance. Patient stakeholders and patients' families evaluated whether the OPD staff always provides clear information, are friendly and polite, and open services on time. Their appearance is always neat, and they are very good at their field of work. A good, caring attitude and the availability of expert doctors as needed also support services. In addition, stakeholders also evaluated the hospital's cooperation with several institutions or companies, which showed an increase in networking (39.^40^).

**Validity testing**: All 5 of the HSC questionnaires were proven to be valid with an average p = 0,0046 (α = 0.05), and those also proven to be reliable with an average p = 0,995 (α = 0.6). Additionally, all five of the HPF questionnaires were also proven valid and reliable, with an average p for validity of 0,0062 (α = 0.05), and average p for reliability of 0,995 (α = 0.05) (Table 2).

The validated hospital-stakeholder tool results are shown in Table 2, while the blueprint is explained in Table 3. Using a 5-point Likert scale (1 to 5); responses range from strongly disagreeing to totally agreeing. Higher scores, depicted in Table 4, indicate greater stakeholder influence, positive perceptions of hospital performance, and service improvement.

**Table 3:** The final blueprint of the tool

Tool	Domain	Sub domain	Item number				

			Patient	Patient Family	Internal Doctor	External Doctor	Insurance Company
Hospital Stakeholder Collaboration	Stakeholder Identification		1 – 5	1 – 4	1 – 2	1 – 2	1 – 2
			(MCQ)	(MCQ)	(MCQ)	(MCQ)	(MCQ)
			6 – 8	5 – 7	3 – 5	3 – 5	3 – 5
			(OEQ)	(OEQ)	(OEQ)	(OEQ)	(OEQ)
	Interactive Dialogue Commitment	Communication Mechanism	1 – 6	1 – 5	1 – 8	1 – 7	1 – 7
		Information fluency	7 – 12	6 – 12	9 – 10	8 – 10	8 – 9
		Stakeholder expectations	1 – 3	1 – 3	1 – 3	1 – 3	1 – 3
		Stakeholder motivation	4 – 6	4 – 6	4	4	4 – 5
	Planning	Stakeholder role	1 – 5	1 – 5	1 – 3	1 – 3	1 – 2
		Stakeholder one goal	6 – 7	6 – 8	4 – 6	4 – 6	3 – 5
	Implementation	Stakeholder collaboration	1 – 4	1 – 4	1 – 4	1 – 4	1 – 4
		Stakeholder coordination	1 – 3	1 – 3	1 – 3	1 – 3	1 – 3
		Stakeholder trust	1 – 7	1 – 7	1 – 6	1 – 6	1 – 7
Hospital Performance Factors	Stakeholder perspective	Service waiting time	1 – 3	1 – 3	1 – 3	1 – 3	1 – 3
		Stakeholder satisfaction	4 – 11	4 – 11	4 – 9	4 – 9	4 – 11
	Financial perspective	Ease of payment process	1 – 2	1 – 2	1	1	1
		Determination of service fees	3 – 4	3 – 4	2	2	2 – 3
	Internal business process perspective	Service flow speed	1 – 4	1 – 4	1 – 2	1 – 2	1 – 3
		The ability of officers to provide services	5 – 6	5 – 6	3	3	4
		Quality of service	7 – 11	7 – 11	4 – 7	4 – 7	5 – 8
		The quality of provision of facilities and infrastructure	12 – 16	12 – 16	8 – 11	8 – 11	9 – 13
		Speed of provision of facilities and infrastructure	17	17	12	12	14
	Staff and Organization Capacity perspective	Skill upgrade	1 – 8	1 – 8	1 – 5	1 – 5	1 – 10
		Network enhancement	9	9	6	6	11

**Table 4:** Operational definition

Tool	Domain	Items	Minimum – Maximum Score	Interpretation
Hospital Stakeholder Collaboration	Patient	47	47 – 235	The higher the score, the higher patient's influence to the hospital
	Patient Family	47	47 – 235	The higher the score, the higher patient family's influence to the hospital
	Internal Doctor	38	38 – 190	The higher the score, the higher internal doctor's influence to the hospital
	External Doctor	38	38 – 190	The higher the score, the higher external doctor's influence to the hospital
	Insurance Company	38	38 – 190	The higher the score, the higher insurance company's influence to the hospital
Hospital Performance Factors	Patient	41	41 – 205	The higher the score, the better patient's perception to the hospital's performance
	Patient Family	41	41 – 205	The higher the score, the better patient family's perception to the hospital's performance
	Internal Doctor	33	33 – 165	The higher the score, the better internal doctor's perception to the hospital's performance
	External Doctor	29	29 – 145	The higher the score, the better external doctor's perception to the hospital's performance
	Insurance Company	39	39 – 195	The higher the score, the better insurance company's perception to the hospital's performance

**Operating procedure for implementation of the hospital's stakeholder collaboration module**: The module, designed for a maximum of 8 weeks, focuses on enabling hospitals to collaborate effectively with stakeholders. It targets quality improvement staff and OPD nurses, imparting knowledge about hospital stakeholder collaboration and guiding them to implement stakeholder collaboration domains within the specified time frame. Utilizing the HSC tool, the domains of hospital-stakeholder collaboration are assessed. This module includes focused group discussions involving hospital management, staff, and stakeholders to identify and prioritize stakeholder needs, leading to actionable strategies. It serves as a platform to foster collaboration and prompt improvements in hospital performance through stakeholder engagement.

The collection of stakeholder data involves a need assessment activity and directly engaging hospital staff in interacting with patients. The hospital's quality staff's and OPD nurses conduct this assessment, which spans 8 weeks and entails mapping potential stakeholders and inviting them to participate. This process involves information gathering across three sessions in the initial three weeks and an additional session in the eighth week for evaluating service improvements. After the assessment, a questionnaire is followed by focused group discussions to formulate a Plan of Action (POA), which guides five weeks of service improvements. In the eighth week, stakeholders evaluate service enhancements based on domains outlined in the HPF questionnaire.

## Discussion

The result showed that valid and reliable HSC and HPF tools have been developed. Both tools have been compiled in a module for nurses or quality improvement staff to use in the OPD setting. The module has been tested as valid and reliable for five different stakeholders: patients, patient families, internal doctors, external doctor, and the insurance company. A similar tool to assess satisfaction for hospital care (SHCQ) according to staff and patients of hospital wards has been developed and tested, and it has been shown to be very reliable (37). Another developed tool to assess the Quality of Interactions Schedule (QuIS) between patient and staff interaction in the acute care setting showed a good level of agreement (38). The HSC and HPF tools have two differences, first: they are developed for the OPD setting, and second: the assessment of service improvement goes through the identification, interview, and FGD processes. The tool was designed this way to achieve a comprehensive assessment of patient needs; it also allows the interaction between the stakeholders who provide service improvement recommendations and the hospital management (4,7,29).

The module recommended that the service improvement project go on for 8 weeks. The stakeholder identification process lasted for 1 week, continued with need assessment for 1 week, and in the third week, there was an FGD with all stakeholders and agreement on the point of action. The time commitment required for quality improvement depends on the project at hand. While some projects may only take a few weeks, others can take several months or longer (39). To put the module into context, it is compared with the Plan-Do-Study-Act (PDSA) cycle. The PDSA has no fixed method on how long the process is; it is rather pragmatic, in that the cycle could be introduced initially to the service improvement process or during the process (40). The study utilized an 8-week duration to implement the service improvement initiative, allowing ample time for assessment (39,41). This time frame is perceived as balanced, offering sufficient duration for evaluation without being too long. The tool involves post-improvement stakeholder evaluation (HPF tool), making the 8-week period reasonable (42). Additionally, the OPD patient in the Indonesian context returns to the hospital regularly every month. This resulted in the patient experiencing the service improvement twice during the service improvement process to generate patient satisfaction, which in turn will increase the patient's trust and commitment to join the evaluation phase (41,42).

The selection of hospital staff stakeholders focused solely on physicians due to the belief that patients seek healthcare services primarily based on their preference for a specific doctor's care. Aside from the health facility's reputation, the patient also expressed the high relevance of the physician as the driver of their visit. Moreover, in a context like Indonesia, where doctors determine the timing of patients' transitions to subsequent care, it becomes rational to exclusively engage physicians in service improvement efforts (13). Research asserts that physicians wield significant influence over the service improvement process, as the hospital's dynamics of illness and treatment center around the patient-doctor relationship. Regrettably, doctors' involvement in the service improvement process remains below 35% due to various constraints such as limited time, challenges to physician autonomy, financial disincentives, and inadequate support for quality improvement initiatives (39). A study by Setyawan, et. al., (2022) supports this phenomenon. The study found that physician-organizational commitment supported the patient satisfaction at 80%. The organizational commitment is crucial to implement holistic and comprehensive care (29).

The HSC and HPF tools were devised to enhance hospital services by delving into stakeholder perceptions and values. This study examines whether these tools effectively capture stakeholder engagement and insights for improved healthcare delivery. Collectively, these studies underscore the centrality of stakeholder collaboration in driving service improvement, aligning with the core tenets of the HSC and HPF tools. These tools facilitate translating stakeholder feedback into actionable improvements by analyzing patient experience data, capitalizing on patient insights, and embracing multifaceted feedback sources (43). The tools utilize FGD between the stakeholder and the hospital management as a means of knowledge management for the hospital management to reduce common barriers such as organizational politics, lack of attention to results, and dissemination (7,20). Significantly, stakeholder collaboration extends beyond engagement, generating transformative care processes and structural outcomes. The incorporation of patient, caregiver, and family viewpoints leads to meaningful changes in healthcare organizations, embodying patient-centered care principles (33,44).

The strength of this research is the development of service improvement tools for OPD settings, where no research has been found to make specific tools for OPD. In addition, this tool also comprehensively examines the needs of five stakeholders. The existing tools, mostly examine the interaction between patients and doctors. The limitation of this research is that it was only carried out in one hospital in Indonesia; so, its application to other hospitals or other countries needs to pay attention to similarity of the hospitals.

The module, which contains the HSC and HPF tools, is a newly developed tool for service improvement in hospital outpatient departments. This module is useful to improve service in the Indonesian hospital. Additionally, this tool adds to the body of knowledge about the available service improvement tools. The module could be studied further by employing in in different outpatient departments and hospitals. A multicenter study to assess the discrepancy between different stakeholder characteristics is also useful.

The objective of this research was to create the Hospital-Stakeholder Collaboration Tool (HSC) Tool and the Hospital Performance Factor Tool (HPF) Tool in order to investigate how stakeholders perceive and value improvements in hospital services. In conclusion, the module containing HSC and HPF tools is valid and reliable to be used in an OPD setting. The module employed 8 weeks of quality improvement for the patient, patient family, internal physician, external physician, and insurance company. The patient and patient family eligible to be stakeholders should actively use the OPD. The physician is the only hospital staff member involved due to the interaction with patients regarding care seeking. Hospital-stakeholder collaboration can positively impact hospital performance across several dimensions. By involving stakeholders in the hospital need assessment process and decision-making, hospital administrators can gain a better understanding of stakeholder needs and priorities, leading to the development of more patient-centered services.
